# UNMASKING THE CONSEQUENCES OF THE COVID-19 PANDEMIC ON COGNITION AND 24-HOUR BEHAVIORS: INSIGHTS FROM THE CLSA

**DOI:** 10.1093/geroni/igae098.2343

**Published:** 2024-12-31

**Authors:** Ryan Falck, Teresa Liu-Ambrose, Megan O’Connell, Eric Smith, Lauren Griffith, Benoit Cossett, David Hogan, Christina Wolfson

**Affiliations:** University of British Columbia, Vancouver, British Columbia, Canada; University of British Columbia, University of British Columbia, British Columbia, Canada; University of Saskatchewan, Saskatoon, Saskatchewan, Canada; University of Calgary, Calgary, Alberta, Canada; McMaster University, Hamilton, Ontario, Canada; University of Sherbrooke, Sherbrooke, Quebec, Canada; University of Calgary, Calgary, Alberta, Canada; McGill University, Montreal, Quebec, Canada

## Abstract

An unintended side-effect of the COVID-19 pandemic has been changes in lifestyle factors which impact middle-aged and older adult cognition – including changes in 24-hour behaviours (i.e., physical activity, sedentary behaviour, and sleep). In a longitudinal analysis of the Canadian Longitudinal Study on Aging (CLSA) tracking cohort, we explored age- and sex-differences in the effects of the COVID-19 pandemic on cognition and 24-hour behaviours, and whether pandemic-related changes in 24-hour behaviours and cognition are associated. We included cognitively healthy participants at baseline (2012-2105), follow-up 1 (FU1; 2015-2018), and follow-up 2 (FU2; 2018-2021), with complete neuropsychological testing data (N=11,355). Cognition and 24-hour behaviours were indexed at each timepoint. Participants were categorized into pre-pandemic (N=6,174) and post-pandemic (N=5,181) cohorts based on whether FU2 assessments occurred before or after COVID-19 pandemic onset (March 11th, 2020). We examined time x cohort changes in cognition and 24-hour behaviours from FU1 to FU2, and if changes in 24-hour behaviours from FU1 to FU2 were associated with changes in cognition. All models were allowed to vary by age and sex. Our results indicated that post-pandemic cohort males and females aged 65+ years had significantly worse cognition and poorer 24-hour behaviours from FU1 to FU2 than their peers in the pre-pandemic cohort (p’s< 0.05). However, changes in 24-hour behaviours from FU1 to FU2 were unassociated with changes in cognition, irrespective of age or sex. Our results highlight that the pandemic negatively impacted 24-hour behaviours and cognition in older adults – although these effects may be unassociated with each other.

